# Evolution of the Swiss pork production systems and logistics: the impact on infectious disease resilience

**DOI:** 10.1038/s41598-025-92011-x

**Published:** 2025-03-06

**Authors:** Francesco Galli, Saskia Perret-Gentil, Antoine Champetier, Rita Lüchinger, Myriam Harisberger, Thibault Kuntzer, Stefan Rieder, Christina Nathues, Beatriz Vidondo, Hartmut Lentz, Vitaly Belik, Salome Dürr

**Affiliations:** 1 Veterinary Public Health Institute, University of Bern, 3097 Liebefeld, Switzerland; 2 Graduate School for Cellular and Biomedical Sciences, University of Bern, 3012 Bern, Switzerland; 3 Swiss 3R Competence Center, 3012 Bern, Switzerland; 4 Pig Health Services SUISAG, 6204 Sempach, Switzerland; 5 Identitas AG, 3014 Bern, Switzerland; 6 Federal Food Safety and Veterinary Office, 3097 Liebefeld, Switzerland; 7 Institute of Epidemiology, Friedrich-Loeffler-Institute, 17493 Greifswald, Germany; 8 Institute of Veterinary Epidemiology and Biostatistics, Freie Universität Berlin, 14163 Berlin, Germany

**Keywords:** Livestock production systems, System evolution, Swine infectious diseases, Disease surveillance, Disease resilience, Infectious diseases, Epidemiology, Risk factors

## Abstract

Livestock production systems are complex and evolve over time, affecting their adaptability to economic, political, and disease-related changes. In Europe, disease resilience is crucial due to threats like the African swine fever virus, which jeopardizes pork production stability. The European Union identifies farm production type as a key risk factor for disease spread, making it important to track changes in farm production types to assess disease risk. However, detailed production type data is often lacking in national databases. For Swiss pig farms, we used prediction and clustering algorithms to classify 9’687 − 11’247 trading farms between 2014 and 2019 by one of eleven production types. We then analyzed the pig trade network and stratified farm centrality measures (ICC and OCC) by production type. We found that 145 farms belonging to three production types have substantially higher ICC and OCC than other farms, suggesting that they could be the target of disease surveillance programs. Our predictions until 2025 show an increase both in overall pig trade network connectivity and in proportion of production types with high ICC and OCC, indicating that the structural changes in the Swiss pig production system may increase infectious disease exposure over time.

**Supplementary Information:**

The online version contains supplementary material available at 10.1038/s41598-025-92011-x.

## Introduction

Livestock production in industrialized countries consists of complex and varied systems, ranging from almost exclusively large operations in some countries, to a mix of big- and small-scale production in others^1^. The diversity of livestock production systems can be expected to have important impacts, for instance in terms of varying resilience to infectious disease^2,3^. The outbreak of African swine fever (ASF) in Europe shows higher losses in terms of pig production in countries with backyard farms compared to countries with industrialized pig production, where the virus is currently almost exclusively affecting wild boar populations^4^.

If different production systems have different levels of infectious disease resilience, it is crucial to track changes in the structure of the production chain and assess whether these changes have the potential to translate into different levels of exposure to disease propagation. In Romania, for instance, a significant drop in the number of ASF cases within the domestic pig population occurred in parallel to a large decrease in the number of pig holdings with fewer than 100 pigs^4^.

The maximum number of animals kept by Swiss pig farms is limited by legislation. This leads to an average of 241 pigs in Swiss farms, which represents 30% of an average German premise and only 5% of a Danish one^5^. In addition, although Switzerland is geographically located in Europe’s center, it is isolated in terms of pig trade from the European Union^6^. The trade isolation gives the country a strategic advantage in terms of health in the pig population^7^. This results in the maintenance of a high health status in the Swiss pig population, despite the small scale of farming. For instance, from 1996 to 2003, sanitation control programs led to a drastic reduction of enzootic pneumonia and *Actinobacillus pleuropneumoniae*infections^8^. These two diseases, in addition to others such as PRRS, are prevalent in neighboring countries, but absent or consistently under control in Switzerland. Nonetheless, preparedness for infectious diseases, currently with ASF as a focus, has a high priority in order to maintain the high health status. The elevated wild boar density both in regions bordering other countries and in areas with extensive pig production exposes the country to infectious disease introduction^9^. Here, the important heterogeneity in farm types, ranging from highly specialized to highly diversified, leading to intensive pig trade between farms, may favor the spread of disease once introduced in the pig production system^10^. While the production type of farms may be an important factor for disease spread, to date the information on production types cannot be found in any national database, as it is the case in most countries.

In countries with comprehensive trade recording systems, network analysis of livestock trade has been extensively used in the last decade to evaluate infectious disease spread potential between farms^11–17^. Two centrality measures have been particularly considered to either identify sentinel farms to put under disease surveillance, or to estimate the maximum epidemic size in case of disease incursion: ingoing contact chain (ICC), representing the number of farms by which a farm is directly or indirectly reached via trade within a given time frame, respecting chronological order of trade contacts^18^, and outgoing contact chain (OCC), representing the number of farms that a farm directly or indirectly reaches via trade within a given time frame, respecting chronological order of trade contacts^19,20^. Since farms with high OCC can spread disease to many farms, and farms with high ICC can contract disease from many farms, putting those farms under surveillance may allow for early detection of epidemics. In Switzerland, Sterchi et al. analyzed the Swiss pig trade network and identified a small number of farms with high ICC and OCC. They discussed how those farms may belong to specific production types, an aspect that deserves further investigation^10^. Differential centrality by production type was also identified in the Swiss cattle trade network^21^.

In the present study, we analyze the Swiss pig trade network and assess the association between production type and farm centrality in the network. We explore the evolution of this association over time between 2014 and 2025, and we discuss its relevance for infectious disease preparedness of the livestock industry, using the example of the Swiss swine sector. As the information on pig farm production type is not systematically collected in Switzerland, as a first step we build random forest models to predict the pig types transported for all transport movements recorded in the official Swiss animal movement database (TVD) between 2014 and 2019. Then, by using the TVD data categorized by pig type, we apply three different algorithms to cluster Swiss pig farms by production type, calculate ICC and OCC for each farm, and finally compare them between pig farm types.

## Results

### Predicting transported pig types and clustering farms by production type

With the implemented random forest models, we were able to classify all TVD from 2014 to 2019 into seven pig types: boar, gilt, breeding sow in segmented piglet production ring, 10-kilogram piglet, 25-kilogram piglet, finisher pig, and old breeding pig. The overall weighed accuracy of predictions was 99.1%, and overall weighed F1-Score was 95.9%. Given the very high level of accuracy of our predictions, we used the transported pig type information to cluster Swiss pig farms into production types.

**Fig. 1:**
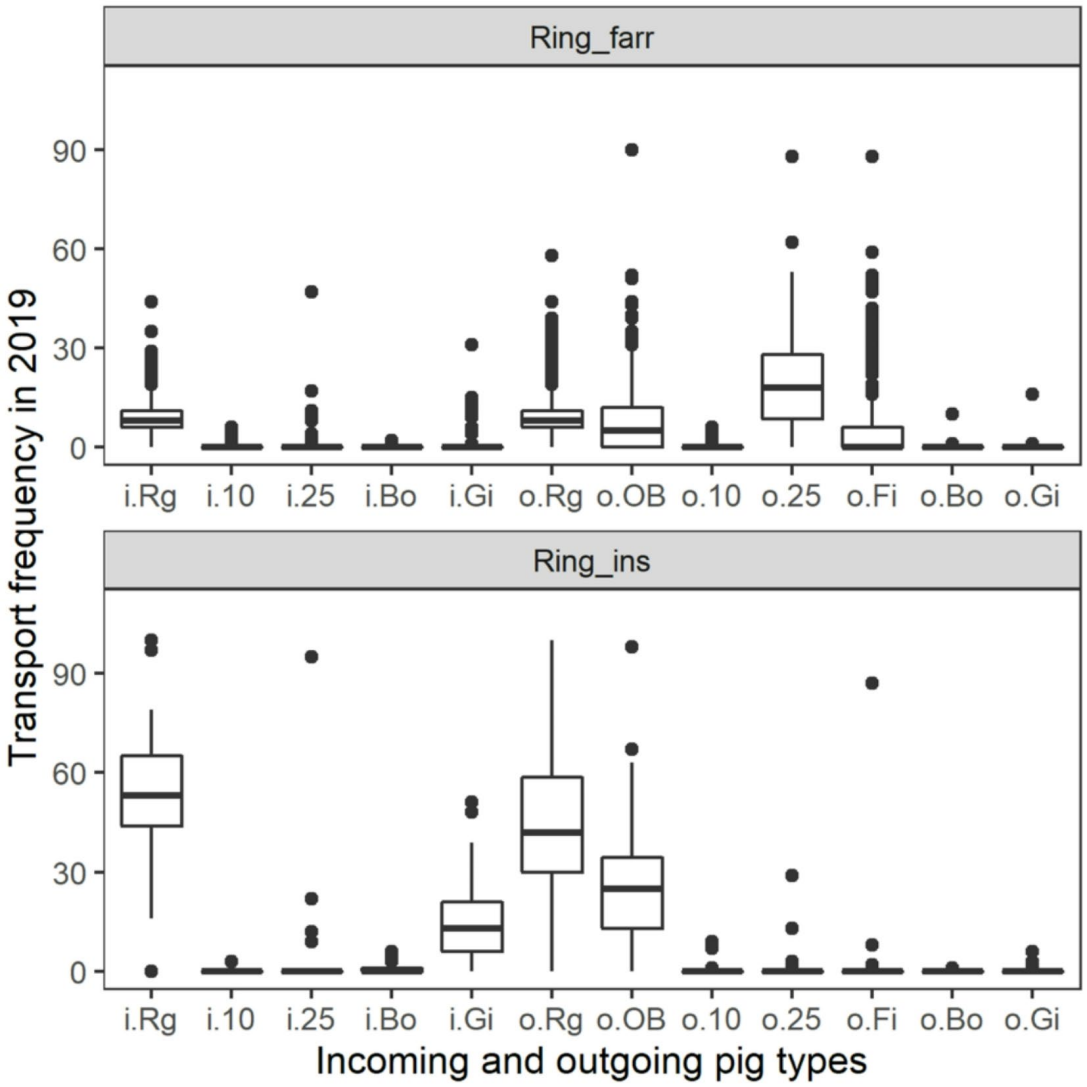
Two examples of cluster “fingerprints” for the clusters *Ring_farr* and *Ring_ins*, obtained by plotting boxplots of incoming and outgoing frequency of transport movements by pig type in 2019. Movement categories: “i.” = incoming, “o.” = outgoing, “Rg” = sows in a segmented piglet production ring, “10” = 10-kilogram piglets, “25” = 25-kilogram piglets, “Bo” = boars, “Gi” = gilts, “OB” = old breeding pigs, “Fi” = finisher pigs.

Among the three clustering algorithms used to identify pig farm production types, the one that performed the best was Partitioning Around Medoids (PAM)^22^ with nine clusters (*k* = 9), followed by k-means with *k* = 12 and PAM with *k* = 12. After discussion of cluster composition with Swiss pig production experts, we concluded that the PAM clustering with *k* = 11 would most realistically describe the Swiss pig production system. Figure 1 shows two examples of boxplot fingerprints with the frequency of incoming and outgoing transport movements by pig type. Boxplot fingerprints for all 11 production types can be found in the Supporting Figure S1.

**Fig. 2:**
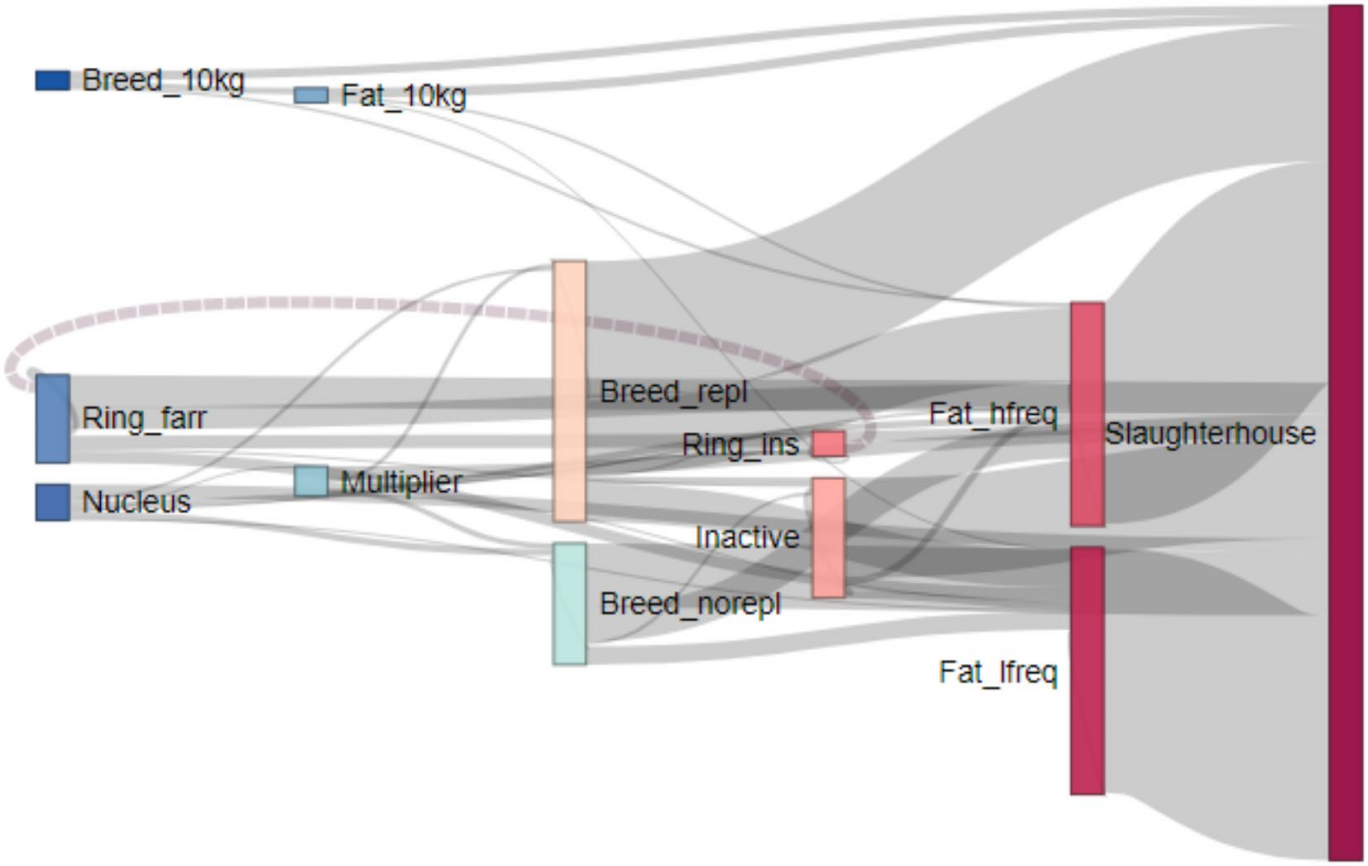
Alluvial plot displaying pig transport movements flows in 2019 between and within pig production types, as well as to slaughterhouses. For visualization purposes, movement flows of less than 250 between production types were excluded. *Nucleus* farms do not have any incoming transport movements and mainly send out gilts to either multiplier herds or breeding farms. *Multiplier* farms receive gilts from nucleus herds, multiply them and send the produced gilts to breeding farms. Breeding farms are *Breed_repl* and *Breed_norepl. Breed_repl* farms have fewer incoming transport movements than *Breed_norepl* farms, as *Breed_repl* farms do gilt replacement on-farm instead of receiving new gilts from other farms. Breeding farms typically send piglets to fattening farms once they weigh 25 kg. Instead, *Breed_10kg* already ship piglets out when they reach a weight of ten kilograms, to *Fat_10kg* farms which cover this intermediate fattening step. *Fat_10kg* farms then either send 25-kilogram piglets to standard fattening farms (*Fat_lfreq* and *Fat_hfreq*) or they fatten piglets until they reach slaughter weight and send them directly to slaughter. *Ring_farr* farms send their sows to *Ring_ins* farm, where insemination takes place. The sows then return to *Ring_farr* farms where they give birth and wean the piglets. In the figure, this back flow from *Ring_ins* to *Ring_farr* farms is represented by a dashed line. The piglets weaned in *Ring_farr* are then shipped to fattening farms. *Fat_hfreq* and *Fat_lfreq* farms both fatten pigs and ship them on a high and low frequency, respectively, to slaughterhouses when finished. While *Fat_hfreq* farms have almost the same amount of incoming and outgoing pigs, *Fat_lfreq* farms have fewer numbers of incoming pigs than outgoing pigs. This indicates that they also perform some on-site breeding. The *Inactive* cluster is constituted by farms only trading very few pigs within a year.

Table 1 summarizes the distribution of production types of the 9’687 pig farms active in 2019 and their characteristics in terms of pig trade. The overall picture resulting from our farm classification is a production system composed of few nucleus and multiplier herds (*Nucleus* and *Multiplier*), two types of breeding farms (*Breed_repl* and *Breed_norepl*), two types of fattening farms (*Fat_hfreq* and *Fat_lfreq*), two farm types performing an intermediate step between breeding and fattening (*Breed_10kg* and *Fat_10kg*), two types of farms forming segmented piglet production rings (*Ring_farr* and *Ring_ins*), and a large group of farms only trading a few times a year (*Inactive*). A detailed description of all production types is provided as Supporting Text in the Supporting Information (SI) Appendix. The alluvial plot in Fig. 2 visually represents the trade flow between and within production types, and from production types to slaughterhouses. It also allows to evaluate the plausibility of the clusters generated using the chosen clustering algorithm. Nucleus and multiplier herds are found at the beginning of the production chain, shipping gilts and boars to all the farm types performing breeding. Breeding farms do the farrowing and subsequently send piglets to fattening farms, where they reach slaughter weight and are transported to slaughterhouses. Beside the standard production chain, Fig. 2 displays the intensive trade between segmented piglet production farms, as well as the parallel production chain of *Breed_10kg* and *Fat_10kg* farms, where piglet transfer takes place at 10-kilogram bodyweight (instead of 25-kilogram). Finally, the figure shows that in Switzerland all production types, not only fattening farms, perform fattening in some measure and ship fattened pigs to slaughterhouses. An alluvial plot without transport movements to slaughterhouses can be found as Figure S2 in the SI Appendix and provides a clearer visualization of transport movement flows between production types.

When looking at cluster stability over a 3-year period from 2017 to 2019, out of the 8’111 farms present in the TVD during that whole period, 6’159 (75.9%) were classified in the same cluster for every one of the three years. As it is not plausible for a farm to change its production type back-to-back two years, we calculated the proportion of farms having the same classification in 2017 and 2019, but a different classification in 2018, as potential misclassifications. Looking at this metric, we found that out of the 8’111 farms, only 457 (5.6%) were classified differently in 2018 than in 2017 and 2019, which increased the confidence in the used methodology of production type identification. Table S1 in the SI Appendix reports this proxy error rate stratified by cluster.

### Farm centrality metrics in the transport network

**Fig. 3:**
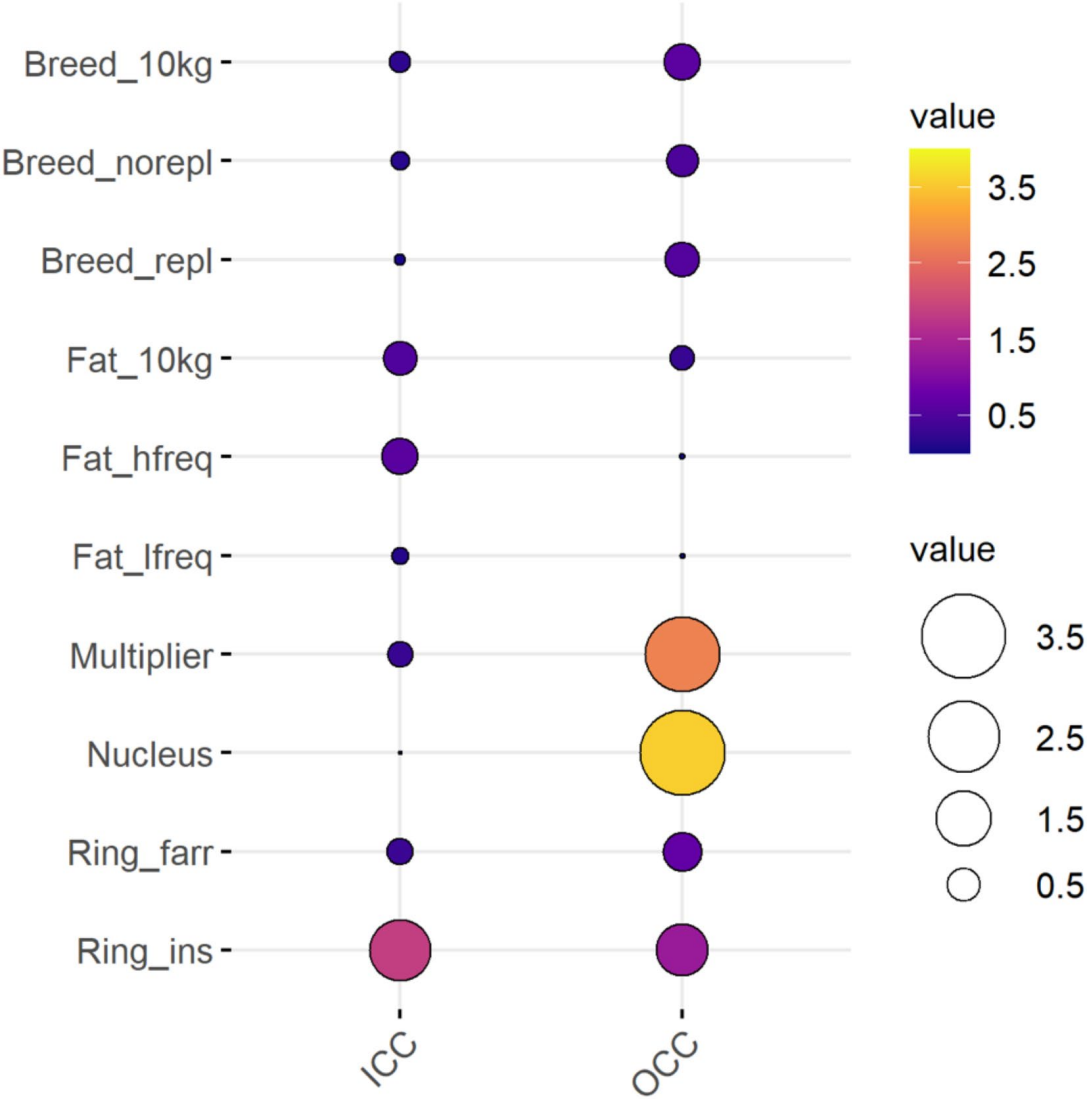
Balloon plot showing average weekly ICC and OCC values in 2019, stratified by production type. Circle size is a continuous representation of ICC and OCC values, while the color scale is a discrete representation for values 0.0 < 0.5, 0.5 < 1.5, 1.5 < 2.5, 2.5 < 3.5 and higher than 3.5.

Among the 4’362 farms that were not classified as *Inactive* in 2019, 45’521 transport movements took place. Given the low relevance of farms in the *Inactive* cluster in terms of infectious disease propagation via pig trade, they were excluded from the analysis of the transport network. Overall average weekly ICC and OCC in the 2019 Swiss pig transport network were 0.264. This means that, on average in any given 7-day period within 2019, a pig farm could directly or indirectly reach and be reached by 0.264 pig farms through trade. Figure 3 shows cluster-specific average weekly ICC and OCC values. The importance of looking at stratified ICC and OCC values derives from the fact that a small number of farms with higher ICC and/or OCC values than average could be targeted for infectious disease surveillance strategies. The *Fat_10kg*, *Fat_hfreq* and *Ring_ins* clusters, amounting to 888 farms combined, had substantially higher values than overall average weekly ICC, respectively 0.529, 0.621 and 1.861. Much higher than overall average weekly OCC was found among the 98 farms belonging to the *Multiplier*, *Nucleus* and *Ring_ins* clusters, respectively 2.761, 3.578 and 1.295. The *Fat_lfreq* cluster (2’010 farms) had very low network metrics, i.e., an ICC of 0.127 and an OCC of 0.007. ICC and OCC values for all clusters are reported in Table S2 in the SI Appendix.

### Time trends of production types and farm centrality

**Fig. 4:**
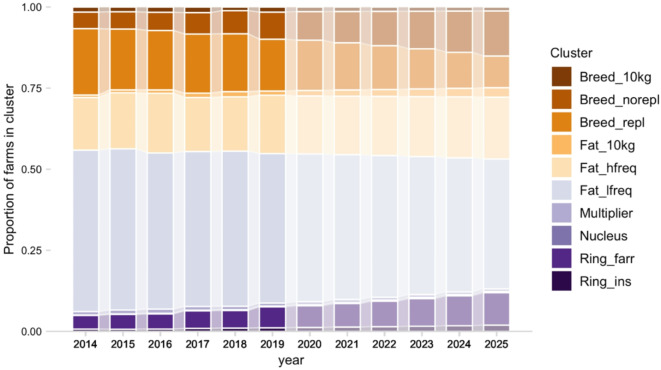
Stacked bar plot showing the proportion of farms in each production type compared to the total number of farms in a year, for the period 2014–2025. Each production type is identified by a color code shown in the legend. Predicted values for the period 2020–2025 are indicated by bars with lower color opacity.

Figure 4 depicts the annual proportion of farms in each production cluster in the 2014–2025 period, observed for 2014–2019 and linearly predicted for 2020–2025. Overall, a shift can be observed from less to more specialized farms. The proportion of farms in the *Breed_norepl* cluster is forecasted to increase from 5.1% in 2014 to 13.9% in 2025, and the proportion of farms in *Breed_repl* to decrease from 20.5% in 2014 to 9.8% in 2025. A less pronounced shift is observed between the two fattening clusters, i.e., *Fat_hfreq* (from 16.2 to 19.1%) and *Fat_lfreq* (from 49.7 to 40.1%). An almost 2.5-fold increase in the proportion of ring farms (*Ring_farr* and *Ring_ins*) is predicted in 2025 (11.9%) compared to 2014 (4.9%). All proportions (with 95% confidence intervals for years 2020–2025) are reported in Supporting Dataset S1.

**Fig. 5:**
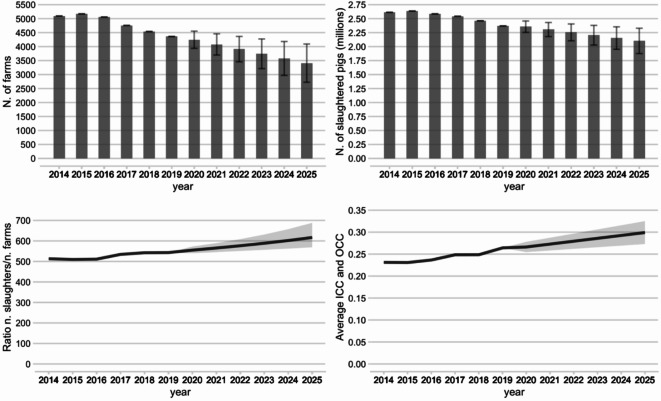
Time trends relative to the Swiss pig production system for the period 2014–2025. From top left to bottom right: number of farms; number of slaughtered pigs in millions; ratio of number of slaughters vs. number of farms; average weekly ICC and OCC. For the 2020–2025 predicted values, 95% confidence intervals are shown with caps for bar plots and with grey areas for line plots.

Time trends of pig production and of farm centrality metrics are shown in Fig. 5, and values (with corresponding 95% confidence intervals for 2020–2025) are reported in Supporting Dataset S2. The total number of farms (excluding *Inactive* farms) strongly decreased from 5’094 in 2014 to 4’362 in 2019 (14.4% decrease) and is predicted to become as low as 3’408 in 2025 (33.1% decrease compared to 2014). In parallel, a 9.2% decrease in the number of slaughtered pigs happened between 2014 (2.61 millions) and 2019 (2.37 millions), and a 19.6% decrease is predicted between 2014 and 2025. As the decrease in the number of slaughtered pigs is less pronounced than the decrease in the number of pig farms, the number of slaughtered pigs/number of farms ratio increased by 5.9% between 2014 and 2019 and is forecasted to increase by 20.2% in 2025 compared to 2014, leading to overall larger farms. In terms of farm centrality in the transport network, average ICC and OCC were 0.231 in 2014, 0.264 in 2019 (14.3% increase compared to 2014) and were predicted to be 0.299 in 2025 (29.4% increase compared to 2014). The rising ICC and OCC values are an indication that the Swiss pig farms are becoming increasingly connected via pig trade.

## Discussion

This work provides for the first time a comprehensive portrait of the Swiss pig production chain and shows how its current and future structure may affect its resilience to infectious diseases. Our farm classification by production type is a powerful tool to identify candidate holdings for disease surveillance and control purposes. We show that it is important to evaluate farms by their production types because production type implies a farm’s incoming and outgoing numbers of pig movements. In addition, we describe the evolution that is unfolding in the pig production chain, which may alert governmental authorities about emerging risks of vulnerability to infectious diseases.

A previous analysis of the Swiss pig trade network highlighted its highly fragmented nature, which mitigates the risk of large-scale disease spread^10^. In our work, we excluded *Inactive* farms, representing 55.0% of the Swiss pig farms, as we considered their impact to be negligible in terms of disease spread through direct trade. We showed that the risk of disease spread among the remaining farms, producing 96% of pigs sent to slaughterhouses in 2019 (Table S3 in the SI Appendix), is in fact remarkable. Our 7-day ICC and OCC values can support the design and implementation of a fast-response disease surveillance system. We also provide 30-day values (Table S4 in the SI Appendix) to enable comparison with other studies. For instance, an analysis of the Swedish pig trade network in 2008 revealed average monthly ICC and OCC ranging between 0.700 and 0.950 ^18^, which is lower than the Swiss range of 0.967–1.179. The production chain of both countries relies on specialized farms sequentially moving pigs forward to the next production step. In Switzerland, an additional production chain exists in which farms of all production types raise piglets and send them to fattening farms, or even fatten them on-site and send them directly to slaughterhouses (see Fig. 2, and Figures S1and S2 in the SI Appendix). These two parallel production chains contribute to the higher average monthly values of ICC and OCC. In a study of the pig trade network of Northern Germany, also consisting of a “parallel chains” system, monthly average ICC and OCC ranged between 1.000 and 1.250, a result comparable to ours^23^. Multiple production pathways may represent an increased risk of infectious disease incursion for fattening farms, but they may also be advantageous in terms of disease resilience: in case of an outbreak in which farms performing intermediate production steps experience trade restrictions, farms from other production types would keep sending piglets to fattening farms and to slaughterhouses.

Our findings are timely in the current development of European policy. In 2020, the European Union declared production type to be an important risk factor for designing livestock disease surveillance and control strategies^24^. In the case of porcine diseases, several studies evaluated the association between disease spread and production type, when the latter information was available^3,13,14,18,25–30^. In other countries where it was not available, it was generated through approximations or prediction models^31–34^. In both cases, pig production type was mostly confined to the broad categories of breeding, breeding-to-fattening and fattening, which limited its use for risk-based disease surveillance and policymaking in general. Brock et al. showed that using animal trade data it was possible to classify Irish cattle farms into as many as 17 distinct production types^35^. The work was later used to describe the evolution of the cattle sector over five years and increase awareness on how this information could be used for very concrete applications, such as monitoring the consequences of the introduction of a law abolishing milk quotas on the structure of the production chain, or designing an intervention for the eradication of bovine herpesvirus type 1^36^. Our stratified network analysis shows that connectivity of Swiss pig farms greatly varies according to the production type they belong to. *Ring_ins* farms and two types of fattening farms (*Fat_10kg* and *Fat_hfreq*) have a weekly average ICC between 2.0 and 7.0 times higher than the overall weekly average ICC. *Nucleus*, *Multiplier*, and *Ring_ins* farms have between 4.9 and 13.6 times higher than overall weekly average OCC. In 2019 the number of farms belonging to those five production types was 986, corresponding to 10.2% of all pig farms. Although higher than average, the ICC of *Fat_10kg* and *Fat_hfreq*, amounting together to 841 farms, is still very low and thus may makes them less relevant for disease surveillance. The remaining 145 farms, belonging to the clusters *Nucleus*,* Multiplier* and *Ring_ins*, and corresponding to 1.5% of all pig farms, may be optimal candidates as sentinel farms in an early outbreak detection system. The addition of *Fat_hfreq* farms with highest ICC may also improve detection performance.

In terms of trends, it appears that the proportion of farms belonging to more specialized production types, such as breeding farms without on-site sow replacement, is rapidly growing. A higher degree of specialization means more pig trade between farms which, in turn, leads to more frequent farm contacts with infectious disease spread potential. A specific cause of concern, given their high ICC and OCC, is that the proportion of piglet production rings (*Ring_ins* and *Ring*_*farr*) is forecasted to increase to 11.9% in 2025, compared to 2014 and 2019 proportions of 4.9% and 7.6%, respectively. While these rings are often characterized as a type of sustainable business model^36^, considering the aforementioned risks, and their historical involvement in infectious disease outbreaks^37^, it should be carefully evaluated whether replacing standard piglet-producing farms with ring farms would impact disease resilience.

On the other hand, frequent trading does not always imply higher risks for disease spread. External biosecurity measures can be highly effective. In Switzerland, those farms with higher ICC and OCC, such as *Ring_ins* and *Ring*_*farr*, and even more so *Nucleus* and *Multiplier*, are highly specialized and professionalized farms with enhanced disease awareness and mostly rigorous biosecurity measures, including change of clothes and shoes, and showering stations. Focus would need to be put on raising awareness for proper biosecurity strategies that are followed by everyone entering the farm (trader, veterinarians, feed providers) on all farms within these categories of enhanced disease spread risks, particularly when they evolve to increase in numbers.

The increasing disease propagation risk is reflected in the almost 30% expected growth in average weekly ICC and OCC between 2014 and 2025 according to our predictions. This growth is driven to a great extent by the increase in the proportion of farms belonging to highly connected production types as mentioned in the previous paragraph. The substantial decrease in absolute number of scarcely connected farms plays an equally important role, as the production type with the largest decrease of farms from 2014 to 2019 was *Fat_lfreq* (from 2’534 to 2’010 farms), which is also the production type with lowest average OCC and third lowest average ICC. When removing many of those farms from the trade network, the remaining farms appear more connected on average. The significant drop in number of *Fat_lfreq*farms may be explained by the fact that they are smaller, less commercially competitive holdings with a potentially less economically sustainable business model. While the trend in industrialized countries of a move towards large, commercial livestock farms is a well-documented phenomenon in France, Germany or the United States, the market and policy settings of Switzerland differ in important aspects and result in country-specific incentives to avoid concentration and operation size increases^38^. Overall, the Swiss pig sector, like most other Swiss livestock sectors, is partially protected from direct competition from other countries through import restrictions, significant producer subsidies and other forms of support, as well as a domestic market where domestic origin is valued and rewarded by price premium^39^. These aspects limit the pressure on the livestock sector to constantly increase farm size.

Furthermore, in Switzerland the maximum number of animals per holding is defined by legislation related to animal welfare. A pig premise is allowed to keep up to 250 sows or up to 1500 fattening pigs, which results in smaller farm sizes by far when compared to most European countries^40^. Considering recent initiatives from the Swiss society, it is not foreseen that the importance given to animal welfare over cost considerations will change in the near future^41^. A consequence of the maximal limit of pigs per farm is the creation of «ring farms» that specialize on one step of piglet production, such as the *Ring_ins* and *Ring*_*farr.* By splitting the steps across different farms, operations can limit their effective size with the side benefit of increased specialization. Obviously, this also results in the need for more transport movements as highlighted in our analysis. There is thus a trade-off between higher animal welfare on the farms by limiting the number of animals and higher carbon footprint by the more frequent transports required by this system.

*Fat_lfreq *exhibits the most substantial drop in farm units, however the number of pig farms has been experiencing a decrease overall. The annual number of slaughtered pigs also declined in the 2014–2019 period, and this drop continued in the following years, as shown by our predictions, and as confirmed by national statistics until September 2023 (except for COVID-19 years 2020 and 2021 where meat production slightly increased)^42^. This may be attributed to a lower demand in pork meat in the country^43^, which matches the general trend observed in Europe^44^. However, as the number of slaughters has been decreasing less rapidly than the number of farms, the average number of slaughters per farm has been increasing. Again, this may be due to the drop in numbers of *Fat_lfreq *farms which trade and produce less than other farms, but it could also be attributed to an increase in size of the remaining farms. If farms become larger besides becoming more specialized, they may increase even further their number of contacts with other farms. Additionally, the authors of^45^ simulated the impact of different cattle densities among farms and found that high livestock density favors on-farm disease spread which, in turn, makes further spread to other farms more likely.

Disease spread potential in a livestock trade network can be reduced by achieving any or a combination of the following: (1) reducing the number of farms, (2) reducing the number of transport movements, or (3) ensuring with strict on-farm and between-farm biosecurity that the transport movements taking place do not have a disease spread potential. Our findings show that in Switzerland scenario 1, i.e. a reduction in the overall number of farms, and especially in small-size farms with limited productivity (*Fat_lfreq *farms), is already taking place even in the absence of specific policy changes. Additionally, given the historical importance of small- to medium-sized businesses in Switzerland, the aforementioned preference of the Swiss population for small-sized farms to ensure high animal welfare, and the fact that the current system has so far been able to guarantee a high health level in the national pig population, we believe that reducing the number of farms and, as a consequence, increasing the farms’ size will happen in the near future with a much faster pace than so far observed. We expect interventions towards adjustments in the transport business (scenarios 2 and 3) to be more successful in mitigating the current and future disease propagation risk. Strengthening the existing legal framework that regulates transport requirements for the supply chain of marketable pigs could, in turn, leverage investment in terms of biosecurity and welfare during pig transport movements by the involved actors in the industry, i.e. pig producers and transport companies. For instance, in Galli et al. risky behaviors related to disease spread during pig loading and unloading were identified both on the side of the farmer and on that of the lorry driver. It was proposed that transport companies should ensure regular biosecurity trainings for their drivers, while the government and pig health services companies should put in place risk awareness campaigns targeting farmers and their biosecurity knowledge^46^.

The meaningful results we obtained from our analyses indicate that a high-resolution farm classification is necessary for supporting evidence-based policymaking. Table S1in the SI Appendix shows that cluster error rate, i.e. the frequency of likely incorrectly classified farms, is generally very low, thus strengthening the validity of using our classification for policy. For the three breeding farm categories, the error rate was higher, ranging between 11.7% and 16.9%. This could be attributed to the great variation of specialization level among Swiss farms. Indeed, breeding farms may often switch from doing their own sow replacement, to introducing new sows from other farms, and vice-versa. Incorrect transport movement notification by farmers may also not be excluded. Overall, although no data are available to directly validate our classification, boxplot fingerprints support the validity of the single clusters, and alluvial plots the plausibility of the interaction between clusters via pig trade. The two visual tools facilitated our exchange with experts to confirm and increase the robustness of our classification. The importance of visual tools to elicit expert knowledge in the classification of farms was indeed stressed by Brock et al. when classifying Irish cattle farms^35^. Despite the robustness of the method applied here, options need to be explored to systematically record production type of farms within the national trade recording systems.

A key limitation of our work is that its analysis period reaches 2019 and we projected figures for the following years, with a sometimes high level of uncertainty. While TVD data until the year 2023 would be relatively easy to obtain, the prediction of transported pig types would require obtaining and merging multiple datasets from different sources and institutions, which are more difficult to access. In our opinion, this limitation highlights the urgent need for governmental and private actors, such as trading companies and pig producers, to make agreements to facilitate data exchange, to allow both long-term planning and prompt responses to disease outbreaks. Another limitation of our work is the lack of alternative disease transmission pathways between farms. Indeed, while direct pig trade was found to be the most relevant pathway for the spread of the most dangerous pig pathogens in Switzerland^46^, various modelling efforts have showed that indirect trade-related^10,13,15,25^and non-trade-related^30,47,48^ disease pathways substantially increase transmission potential. The incorporation of such pathways in future network studies would greatly improve their contribution to policy. Finally, non-compliance with pig transport movement notification may bias the results of our analyses. However, as pig transport movement notification is mandatory by law in Switzerland since 2011, we expect a very low proportion of missing transport movement data.

Our work shows the importance of describing a country’s livestock production system, its evolution over time and the possible consequences that such evolution may have. The authors of^49^ argue that this type of information can be used for disease preparedness, for estimating production type-specific economic output, greenhouse gas emission and biomass distribution, thus allowing to move towards more efficient production systems. The methodological framework that we propose in our study may serve as a guide for such further efforts.

## Materials and methods

### Prediction of transported pig types

To be able to cluster farms into production types, it is required to include the information of pig types that farms receive and send during a given year. This information is not yet systematically collected within the TVD. We obtained pig transport movement datasets from two major Swiss pig traders, where transported pig type is systematically recorded for all movements as one of the following seven types: boar, gilt, breeding sow in segmented piglet production ring, 10-kilogram piglet, 25-kilogram piglet, finisher pig, and old breeding pig. We matched the TVD with the two trader datasets for the period 2014–2019, using as match variables the TVD ID of the sending farm, the TVD ID of the receiving farm, and the date of transport movement. 15% of the TVD transport movements were matched with the trader datasets. To obtain the transported pig type information for the remaining 85% of TVD data, we built random forest prediction models using the *randomForest* R package. As predictors, we used the variables present in the TVD for each transport movement, and variables on pig farm characteristics extracted from the Swiss Agrarian Information System database (AGIS). A list of TVD and AGIS variables used as predictors can be found in Supporting Dataset S3. Five pig types (boar, gilt, breeding sow in segmented piglet production ring, 10-kilogram piglet, 25-kilogram piglet) are only moved from farm to farm, while two pig types (finisher pig and old breeding pig) are only moved from farm to slaughterhouse. Moreover, AGIS data was not available for all TVD observations: 6.9% of TVD transport movements did not match with AGIS neither for the sending nor for the receiving farm, and 2.6% of TVD transport movements matched with AGIS for either the sending or the receiving farm, but not for both. Therefore, the predictors extracted from AGIS could not be used for all farms. As a result, we built a total of six random forest models to predict transported pig type for each specific data subset, namely:

- Farm-to-farm movements with AGIS data available for both sending and receiving farms.
- Farm-to-farm movements with AGIS data available only for sending farm.
- Farm-to-farm movements with AGIS data available only for receiving farm.
- Farm-to-farm movements with AGIS data not available for neither sending nor receiving farms.
- Farm-to-slaughterhouse movements with AGIS data available for sending farm 1
- Farm-to-slaughterhouse movements with AGIS data not available for sending farm.

Of the 15% of TVD data that matched with trader data, we used 80% for training the random forest models and 20% for testing them. As metrics for model performance, for each of the six models we calculated model accuracy and F1-score using the *MLmetrics* R package, and we reported them in Supporting Dataset S3. We also reported the overall prediction accuracy for all models, weighed by the proportions of TVD data that were predicted by each model.

### Production type clustering

For each Swiss pig farm and for the year 2019, we extracted annual frequencies of incoming and outgoing pig transport movements from the TVD. Transport movements were stratified by the seven predicted types of transported pigs. We used the frequencies of movements by pig type as input variables for three different clustering algorithms: k-means, Partitioning Around Medoids (PAM) and hierarchical clustering using the *clValid *R package^50^. The package uses Silhouette Width as internal validation measure, as well as average proportion of non-overlap, average distance and average distance between means to evaluate clustering method stability by comparing the original clustering with the clustering based on the removal of one input variable at once. The combinations of best algorithm and optimal number of clusters (*k*) were ranked with the *RangAggreg* function of *clValid*, using the Cross Entropy Monte Carlo algorithm^51^. We re-assessed the plausibility of clusters and the optimal *k* value with the support of Swiss pig production experts, and by producing alluvial plots with the *sankeyNetwork* R package (*networkD3* package suite) showing pig flows between clusters. Once the final *k* was defined, we evaluated the stability of clusters over a three-year period, namely 2017–2019, by selecting farms present during the whole period and by observing the proportions of cluster changes during that period.

### Metrics of farm centrality in the transport network

Applying social network analysis (SNA) techniques, we built the network of pig transport movements using TVD data of all pig farms in Switzerland having at least one transport movement in 2019. We defined pig farms as nodes and individual pig transport movements between a source and a destination holding as links. We excluded movements from farms to slaughterhouses from this analysis, since we are interested in the network’s relevance in disease propagation, and slaughterhouses are often considered epidemiological dead ends for disease spread (assuming that transport trucks are cleaned after each slaughterhouse visit). We calculated farm-specific average ICC and OCC using the *EpiContactTrace *R package^52^, by first computing ICC and OCC for any possible seven-day time window in 2019, and subsequently averaging them over the year. We calculated overall and cluster-specific ICC and OCC.

Measures deriving from SNA are often categorized by whether they give a static or a temporal representation of the network, and by whether they represent centrality (at node level) or cohesiveness (at network level)^53^. Many published SNA works in the field of veterinary epidemiology include both static and temporal measures, and both centrality and cohesiveness metrics^11–21,23,54^. In Switzerland, this type of comprehensive analysis on the pig trade network was published in 2019 in Sterchi et al.^10^. As the cohesiveness of the network as a whole was presented in that work, we did not find it meaningful to include it again in our study. Moreover, the authors showed that static measures of farm centrality largely underestimated a farm’s importance in terms of targeted surveillance, since average monthly maximum ICC and OCC values were found to be, respectively, 13% and 122% higher than their static counterparts, i.e. in-degree and out-degree. Thus, we chose to focus on node level, temporal SNA metrics to identify potential farms for targeted infectious disease surveillance.

### Time trends of production type and farm centrality

We repeated PAM clustering every year for all farms active at least one time in a year, for the period 2014–2019. For every year in that period, we calculated the proportions of farms in each cluster according to the above-described method. We then predicted the proportion of farms in each cluster for the years 2020 to 2025 by means of linear regression using the 2014–2019 values. Similarly, we linearly predicted number of farms, number of slaughtered pigs, ratio of number of slaughters to number of farms, and average weekly ICC and OCC for 2020–2025 using the 2014–2019 values. Predicted 2020–2025 values were reported together with their 95% confidence interval.

**Table 1:** Descriptive table reporting, for each of the eleven Swiss pig production types and overall, 2019 number of farms and proportion compared to the total number of farms, total and median number of outgoing and incoming transport movements, and total and median number of outgoing and incoming pigs.

Cluster name	Farms		Transport movements		Pigs	
	*N*	%	Total (median) out	Total (median) in	Total (median) out	Total (median) in
*Breed_10kg*	69	0.7	3’166 (40)	529 (9)	118’867 (19)	8’127 (9)
*Breed_norepl*	365	3.8	17’744 (47)	2’456 (4)	466’810 (13)	29’653 (4)
*Breed_repl*	696	7.2	37’762 (52)	1’560 (4)	1’013’469 (13)	25’177 (6)
*Fat_10kg*	56	0.6	2’179 (34)	1’365 (18)	85’077 (30)	82’744 (47)
*Fat_hfreq*	785	8.1	32’094 (35)	22’362 (24)	1’143’091 (29)	1’137’222 (42)
*Fat_lfreq*	2’010	20.7	35’653 (14)	12’172 (6)	868’262 (18)	519’882 (35)
*Inactive*	5’325	55.0	17’391 (2)	3’061 (1)	171’088 (4)	57’920 (6)
*Multiplier*	41	0.4	4’727 (88)	679 (16)	77’608 (6)	21’078 (20)
*Nucleus*	10	0.1	5’334 (146)	1 (1)	86’816 (6)	1 (1)
*Ring_farr*	283	2.9	12’788 (42)	2’797 (9)	363’337 (16)	41’769 (12)
*Ring_ins*	47	0.5	3’848 (70)	3’851 (71)	42’840 (8)	41’845 (10)
**Overall**	**9’687**	**100.0**	**172’686 (8)**	**50’833 (4)**	**4’437’265 (15)**	**1’965’418 (30)**

## Electronic supplementary material

Below is the link to the electronic supplementary material.

Supplementary Material 1

Supplementary Material 2

Supplementary Material 3

Supplementary Material 4

## Data Availability

Aggregated data by farm production type is provided within the manuscript or supplementary information files. Because of privacy concerns, farm-level data used in this study are only accessible by the core study team, as defined at the beginning of the study per data sharing agreements signed by the Principal Investigator and the data owners. For data requests concerning this study, please contact the study’s Principal Investigator, Prof. Dr. Salome Dürr, salome.duerr@unibe.ch.

## Footnotes

1 AGIS data is only collected for farms and does not apply to slaughterhouses.
