# Supplementary material for: Evolution of the Swiss pork production systems and logistics: the impact on infectious disease resilience
Source: Sci Rep. 2025 Mar 6;15:7842. doi: 10.1038/s41598-025-92011-x (PMC11885825; doi:10.1038/s41598-025-92011-x)
Supplement: Supplementary file 1 — Supplementary Material 1 [file 41598_2025_92011_MOESM1_ESM.pdf]

Scientific Reports

Supporting Information for

Evolution of livestock production systems and impact on infectious disease resilience: the Swiss example

Francesco Galli*[1 2], Saskia Perret-Gentil[1], Antoine Champetier[1 3], Rita Lüchinger[4], Myriam Harisberger[4], Thibault Kuntzer[5], Stefan Rieder[5], Christina Nathues[6], Beatriz Vidondo[1], Hartmut Lentz[7], Vitaly Belik[8], Salome Dürr[1]

1. Veterinary Public Health Institute, University of Bern, 3097 Liebefeld, Switzerland
2. Graduate School for Cellular and Biomedical Sciences, University of Bern, 3012 Bern, Switzerland
3. Swiss 3R Competence Center, 3012 Bern, Switzerland
4. Pig Health Service SUISAG, 6204 Sempach, Switzerland
5. Identitas AG, 3014 Bern, Switzerland
6. Swiss Federal Food Safety and Veterinary Office, 3097 Liebefeld, Switzerland
7. Institute of Epidemiology, Friedrich-Loeffler-Institute, 17493 Greifswald, Germany
8. Institute of Veterinary Epidemiology and Biostatistics, Freie Universität Berlin, 14163 Berlin, Germany

* **Email:** f.galli@mail.ch

This PDF file includes:

Supporting text
Figures S1 to S2
Tables S1 to S4
Legends for Datasets S1 to S3

Other supporting materials for this manuscript include the following:

Datasets S1 to S3

Supporting Information Text

Description of production type clusters. Production of genetically high-quality breeding sows is undertaken in nucleus and multiplier farms and is concentrated to only 0.5% of the farms in Switzerland. Nucleus farms generate sows by artificial insemination of the nucleus sows raised on-farm. Thus, a clear indication that the farms in the *Nucleus* cluster are nucleus herds is that they do not have any incoming transports and that they mainly send out gilts to either multiplier herds or breeding farms. *Multiplier* farms receive gilts from nucleus herds, multiply them and send the produced gilts to breeding farms. They likely have few incoming transports and many more outgoing transports because they receive gilts from very few nucleus herds and send gilts to many more breeding farms.

The main difference between the two identified breeding farm types is that, while both types of farms ship out large amounts of 25-kilogram piglets to fattening farms, *Breed_repl* farms have, in proportion, far fewer incoming transports than *Breed_norepl* farms. This is because the *Breed_norepl* farms, which are twice less frequent than the *Breed_repl* farms, do gilt replacement on-farm instead of receiving new gilts from other farms.

Breeding farms typically send piglets to fattening farms once they weigh 25 kilograms. Few farms ship piglets out when they reach a weight of ten kilograms. These farms are represented by the *Breed_10kg* production type, and they mainly send piglets to farms covering this intermediate fattening step, i.e., the *Fat_10kg* cluster.

Ring_farr and *Ring_ins* farms correspond to farms belonging to a piglet production model typical of the Swiss pig farm system, i.e. the segmented piglet production. *Ring_farr* farms send their sows to *Ring_ins* farm, where insemination takes place. The sows then return to *Ring_farr* farms where they give birth and wean the piglets. These interactions explain why *Ring_ins* farms have almost the same number of incoming and outgoing pigs and transports, while *Ring_farr* farms have many more outgoing pigs and transports, as the newly born piglets are subsequently sent to fattening holdings. *Ring_ins* farms are less frequent than *Ring_farr* farms. The data show that they typically offer insemination services to an average of five *Ring_farr* farms each.

Finally, farms in the *Fat_hfreq* and *Fat_lfreq* clusters both have as main activity the fattening of pigs that will be later sent to slaughter. *Fat_hfreq* have almost the same number of incoming and outgoing pigs, which indicates that they almost exclusively perform fattening. Although *Fat_lfreq* farms are 2.5 times more numerous than *Fat_hfreq* farms, they send fewer pigs to slaughterhouses than *Fat_hfreq* farms. Moreover, they have fewer incoming pigs than outgoing. These two facts indicate that these farms are smaller and less specialized fattening farms that also perform some on-site breeding. The *Inactive* cluster is constituted by farms only trading very few pigs within a year. They represent 55.0% of all Swiss pig farms, however they send to slaughterhouses 8 to 11 times fewer pigs than *Fat_hfreq* and *Fat_lfreq* farms.

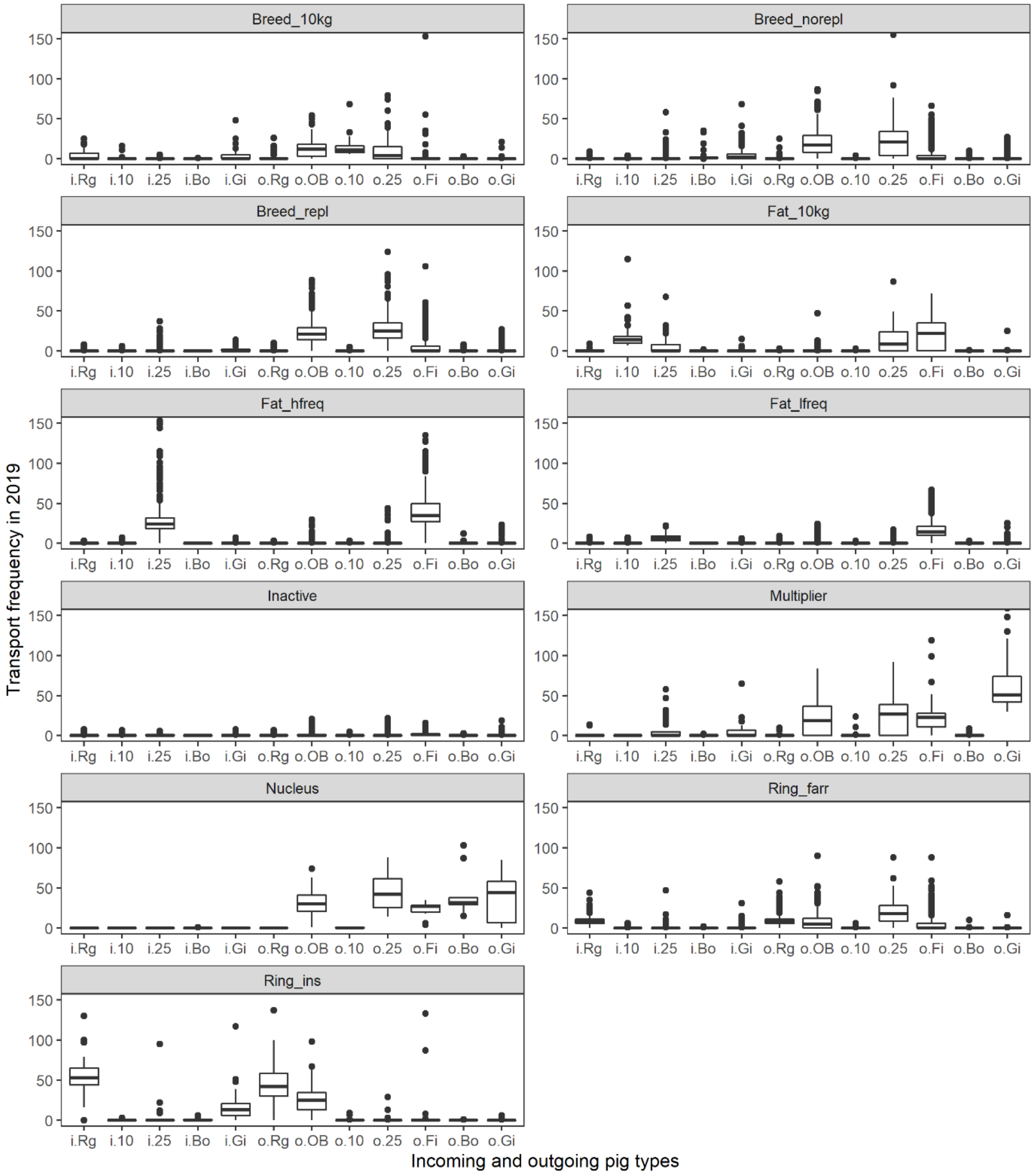

Fig. S1. Cluster "fingerprints" for all farm production type clusters, namely *Breed_10kg, Breed_norepl, Breed_repl, Fat_10kg, Fat_hfreq, Fat_lfreq, Inactive, Multiplier, Nucleus, Ring_farr,* and *Ring_ins*. Cluster fingerprints were obtained by plotting boxplots of incoming and outgoing frequency of transports by pig type in 2019. Transport categories: "i." = incoming, "o." = outgoing, "Rg" = sows in a segmented piglet production ring, "10" = 10-kilogram piglets, "25" = 25-kilogram piglets, "Bo" = boars, "Gi" = gilts, "OB" = old breeding pigs, "Fi" = finisher pigs.

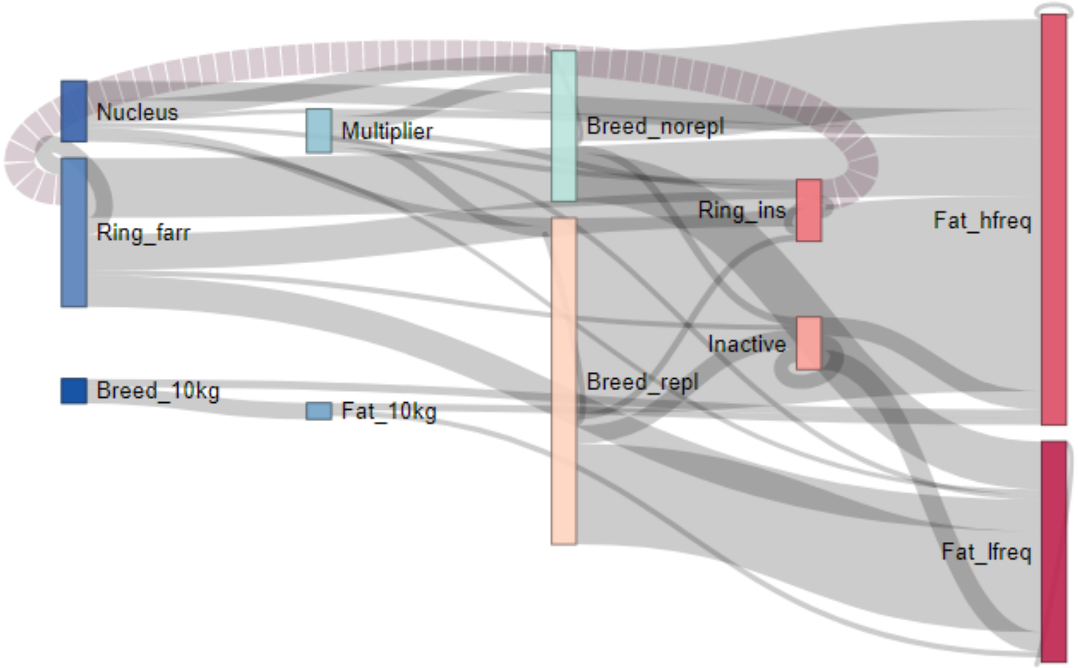

Fig. S2. Alluvial plot displaying pig transports flows in 2019 between and within pig production types. For visualization purposes, transports to slaughterhouses were excluded to provide more zoom into flow between the remaining production types. Also for clearer visualization, transport flows of less than 250 between production types were excluded. For a detailed description of flows between production types, please refer to the Supporting text in this Appendix.

Table S1. Table reporting, for each of the eleven Swiss pig production types and overall, the total number of farms that had trading activities in all three years 2017, 2018 and 2019, the number of farms that were assigned to the same production type cluster in 2017 and 2019, but to a different production type cluster in 2018, and the percentage of such farms among the total number of farms.

Cluster name	N. of farms	N. of farms in same cluster in 2017 and 2019, in different clusters in 2018	%
Breed_10kg	68	8	11.8
Breed_norepl	343	58	16.9
Breed_repl	693	81	11.7
Fat_10kg	53	3	5.7
Fat_hfreq	769	50	6.5
Fat_lfreq	1'975	138	7.0
Inactive	3'835	101	2.6
Multiplier	40	0	0.0
Nucleus	10	0	0.0
Ring_farr	279	17	6.1
Ring_ins	46	1	2.2
**Overall**	**8'111**	**457**	**5.6**

Table S2. Table reporting, for each of the eleven Swiss pig production types and overall, average weekly ICC and average weekly OCC in the Swiss pig transport network in the year 2019.

**Cluster name**	**ICC**	**OCC**
Breed_10kg	0.203	0.643
Breed_norepl	0.155	0.494
Breed_repl	0.046	0.560
Fat_10kg	0.529	0.282
Fat_hfreq	0.621	0.011
Fat_lfreq	0.127	0.007
Multiplier	0.286	2.761
Nucleus	0.002	3.578
Ring_farr	0.317	0.718
Ring_ins	1.861	1.295
**Overall**	**0.264**	**0.264**

Table S3. Table reporting, for each of the eleven Swiss pig production types and overall, and for the year 2019, number of farms, number of pigs sent to slaughterhouses, percentage of pigs sent to slaughterhouses, and the ratio of the number of pigs sent to slaughterhouses over the number of farms.

Cluster name	N. farms	N. pigs sent to slaughterhouse	% of all pigs sent to slaughterhouse	Ratio pigs/farm
Breed_10kg	69	9'873	0.4	143.1
Breed_norepl	365	80'948	3.3	221.8
Breed_repl	696	165'301	6.7	237.5
Fat_10kg	56	41'196	1.7	735.6
Fat_hfreq	785	1'121'305	45.4	1'428.4
Fat_lfreq	2'010	847'189	34.3	421.5
Inactive	5'325	103'935	4.2	19.5
Multiplier	41	22'569	0.9	550.5
Nucleus	10	22'220	0.9	2'222.0
Ring_farr	283	48'671	2.0	172.0
Ring_ins	47	7'709	0.3	164.0
**Overall**	**9'687**	**2'470'916**	**100.0**	**255.1**

Table S4. Table reporting, for each month in the year 2019, average monthly ICC and OCC in the Swiss pig transport network.

Month in 2019	ICC and OCC
January	1.066
February	1.095
March	1.045
April	1.069
May	1.092
June	1.028
July	1.151
August	1.064
September	0.967
October	1.179
November	1.077
December	1.042

Dataset S1 (separate file). For each production type cluster, and for each year in the period 2014-2025, this dataset contains the proportion of farms in the cluster among the total number of farms in that year. It also reports the number of farms in the cluster, together with lower and upper confidence interval values at the 95% confidence level for years 2020-2025, for which numbers of farms were predicted using a linear regression model.

Dataset S2 (separate file). This dataset contains all values plotted in Fig. 5 of the manuscript's main text showing time trends relative to the Swiss pig production system for the period 2014-2025. The sheet "NFarms" reports, for each year in the period 2014-2025, the number of Swiss pig farms excluding farms of the *Inactive* production type cluster. As number of farms for years 2020 to 2025 was predicted using a linear regression model, lower and upper confidence interval values at the 95% confidence level are reported for those years. The sheet "NSlaughters" reports, for each year in the period 2014-2025, the number of slaughtered pigs in Switzerland. As number of slaughtered pigs for years 2020 to 2025 was predicted using a linear regression model, lower and upper confidence interval values at the 95% confidence level are reported for those years. The sheet "Ratio" reports, for each year in the period 2014-2025, the ratio of number of slaughtered pigs in Switzerland over the number of pig farms in Switzerland excluding farms of the *Inactive* production type cluster. As the ratio for years 2020 to 2025 was predicted using a linear regression model, lower and upper confidence interval values at the 95% confidence level are reported for those years. The sheet "ICC_OCC" reports, for each year in the period 2014-2025, the average weekly ICC and OCC in the Swiss pig transport network. As ICC and OCC for years 2020 to 2025 was predicted using a linear regression model, lower and upper confidence interval values at the 95% confidence level are reported for those years.

Dataset S3 (separate file). This dataset contains additional details of the random forest prediction models that we built to predict transported pig types for each transport recorded in the TVD database for years 2014 to 2019. The sheet "Model predictors" contains a list of variables used as predictors in the models. For each predictor we listed predictor name, predictor type (categorical or numeric), a description of the predictor, and from which dataset we extracted the predictor (AGIS or TVD). The sheet "Accuracies and F1-Scores" reports model accuracy and model F1-Score (both reported as percentages) for each of the six random forest models that we built. For each model, we reported: whether it was used to predict pig types for farm-to-farm transports, or for farm-to-slaughterhouse transport; the available data for predictor extraction (TVD, AGIS); model accuracy; model F1-Score; proportion of TVD data predicted by each model. The sheet also reports overall accuracy and F1-Score, calculated as the sum of model-specific accuracies and F1-Scores weighed by the corresponding proportion of TVD data predicted by each model.
